# Case Report: Neurexin-3α-associated autoimmune encephalitis with co-existence of possible anti-MOG-associated involvement

**DOI:** 10.3389/fimmu.2026.1844545

**Published:** 2026-07-01

**Authors:** Rongchuan Li, Zhiqiang Lin, Jinhong Zhuang, Xiaoer Wei, Jingjiong Chen, Xiaofeng Xu

**Affiliations:** 1 Department of Neurology, Jinjiang Municipal Hospital (Shanghai Sixth People’s Hospital Fujian), Quanzhou, China; 2 Institute of Diagnostic and Interventional Radiology, Shanghai Sixth People’s Hospital Affiliated to Shanghai Jiao Tong University School of Medicine, Shanghai, China; 3 Department of Neurology, Shanghai Sixth People’s Hospital Affiliated to Shanghai Jiao Tong University School of Medicine, Shanghai, China; 4 Department of Genetics and Rare Diseases, Shanghai Sixth People’s Hospital Affiliated to Shanghai Jiao Tong University School of Medicine, Shanghai, China; 5 Shanghai Neurological Rare Disease Biobank and Precision Diagnostic Technical Service Platform, Shanghai, China; 6 Neurological Disorder Center, Haikou Orthopedic and Diabetes Hospital of Shanghai Sixth People’s Hospital, Haikou, China

**Keywords:** aseptic meningitis, autoimmune encephalitis (AE), leptomeningeal enhancement, myelin oligodendrocyte glycoprotein (MOG) antibody, neurexin-3α antibody

## Abstract

**Background:**

Autoimmune encephalitis (AE) associated with neurexin-3α antibodies is a rarely reported disorder that commonly presents with severe neuropsychiatric manifestations.

**Case presentation:**

We report a patient who tested positive for neurexin-3α and myelin oligodendrocyte glycoprotein (MOG) antibodies at low titers. The patient presented with seizures, mental and behavioral disturbances, and cognitive impairment. Brain MRI showed increased signal intensity in the bilateral hippocampus and leptomeningeal enhancement in the left cerebral hemisphere. Serum cell-based assay findings, confirmed by tissue-based assay, were positive for neurexin-3α antibodies. The patient was diagnosed with anti-neurexin-3α encephalitis, with possible concurrent anti-MOG-associated involvement. Clinical symptoms improved after treatment with glucocorticosteroids and intravenous immunoglobulin.

**Conclusion:**

Although overlapping antibodies in AE are relatively common, the co-occurrence of neurexin-3α and MOG antibodies has not been previously reported. This case provides new insights into the diagnosis and treatment of neurexin-3α–associated AE.

## Introduction

Neurexin-3α antibody–mediated encephalitis is a newly recognized form of autoimmune encephalitis (AE). Neurexins are synaptic cell adhesion molecules that play important roles in synapse formation and maturation. Antibodies against neurexin-3α can reduce its expression, thereby disrupting synapse formation and potentially affecting presynaptic and postsynaptic functions. A literature review identified 15 reported cases of neurexin-3α encephalitis. This disorder is associated with autoantibodies targeting the extracellular domain of neurexin-3α and is clinically characterized by an infectious-like prodrome, such as headaches and gastrointestinal symptoms, followed by rapid progression to seizures, memory deficits, confusion, impaired consciousness, central hypoventilation, behavioral abnormalities, and speech impairment. Among the 15 reported cases, 10 patients exhibited symptoms suggestive of a preceding infection, including fever, headache, high body temperature, cough, nausea, or diarrhea. In addition, 13 patients developed impaired consciousness and seizures, 4 experienced central hypoventilation, and 2 had severe hallucinations (1, 2). Although some patients respond favorably to immunotherapy, others have poor outcomes, including death. Neurexin-3α antibody–mediated encephalitis is extremely rare, and coexisting positivity for myelin oligodendrocyte glycoprotein (MOG) antibodies has not been previously reported.

In this report, we present a patient with coexisting anti-neurexin-α and anti-MOG antibodies. With the increasing identification of novel autoimmune antibodies and the expanding clinical spectrum of AE, cases with multiple antibody positivity are being reported more frequently. Previous studies have described detailed analyses of patients with concurrent or sequential anti-N-methyl-D-aspartate receptor (NMDAR) and demyelinating antibodies, including anti-MOG IgG and anti–aquaporin-4 (AQP4-IgG) (3). We present this case to enhance understanding of neurexin-3α–associated AE and provide insights into its diagnosis and management.

## Case description

A 26-year-old man was admitted in September 2025 with status epilepticus, along with headache and a one-week history of fever. On emergency admission, physical examination showed a body temperature of 38.5 °C, pulse rate of 90 beats per minute, respiratory rate of 17 breaths per minute, and blood pressure of 120/77 mmHg.

**Figure 1:**
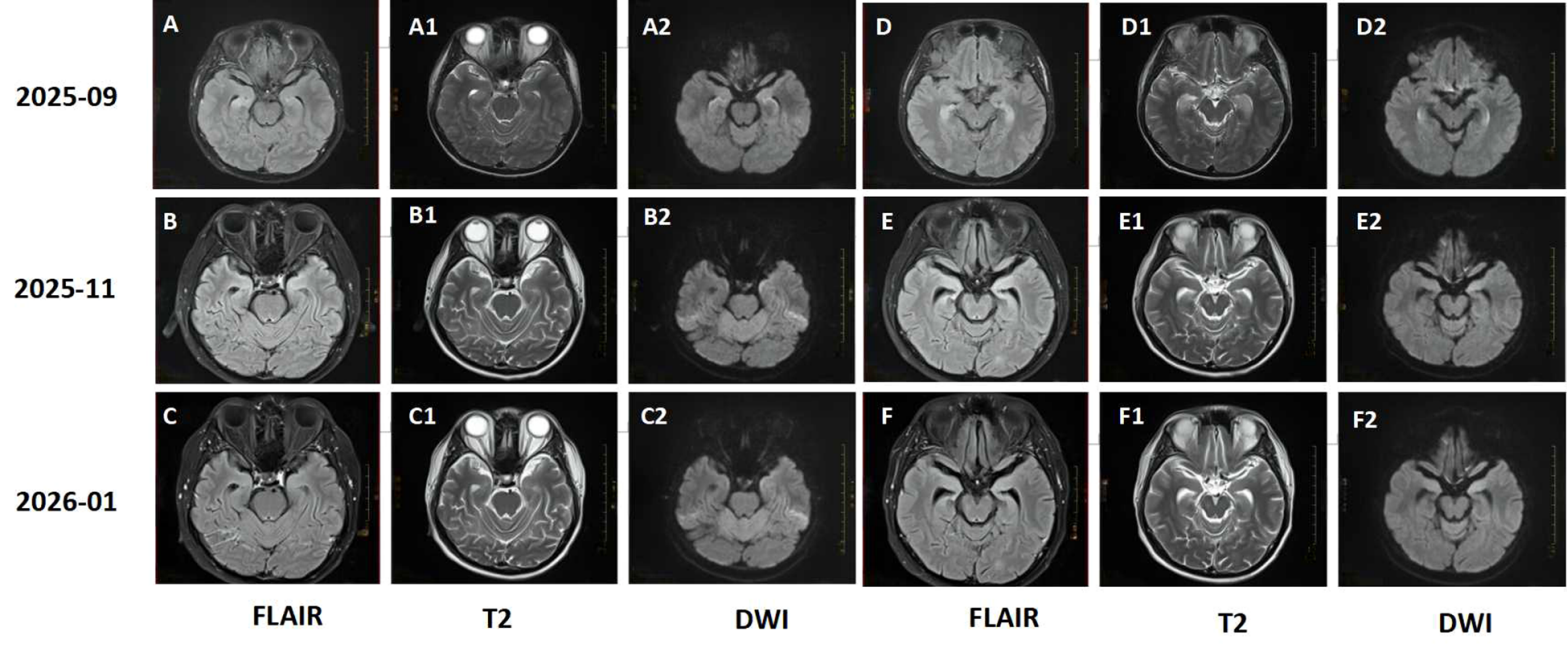
Brain MRI at onset showing abnormal signals in the bilateral hippocampi **(A, D)**. Brain MRI demonstrating bilateral hippocampal alterations with enhancement on FLAIR **(A, D)**, T2 **(A1, D1)**, and DWI **(A2, D2)**.Similar lesions exhibited recovery following 3 and 5 months of treatment. **(B, B1, B2, E, E1, E2, C, C1, C2, F, F1, F2)**.

**Figure 2:**
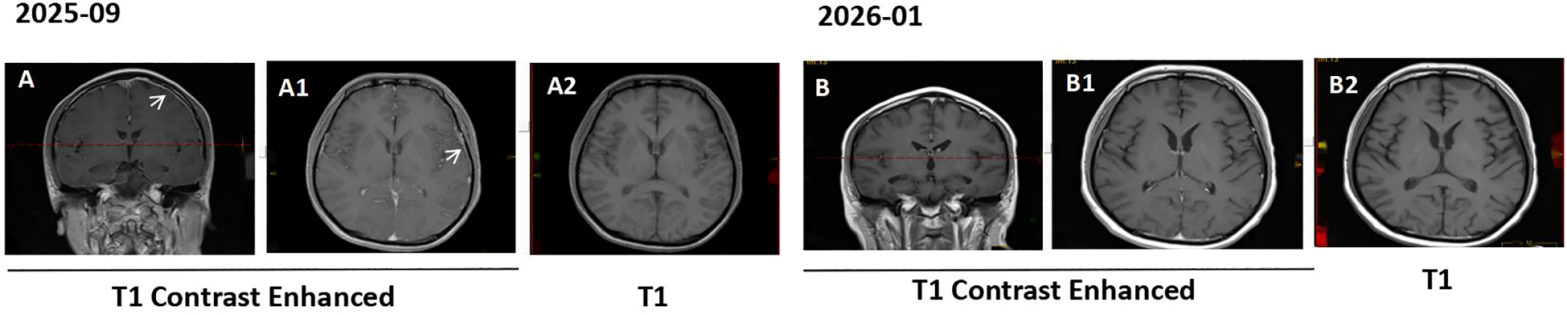
Post-contrast axial T1-weighted imaging **(A, A1)** (**(A2)** T1-weighted imaging) reveals abnormal leptomeningeal enhancement in the left cerebral hemisphere. Follow-up imaging performed 5 months later shows a normal brain appearance and reduced leptomeningeal enhancement in the left cerebral hemisphere on post-contrast axial T1-weighted imaging **(B, B1)** (**(B2)** T1-weighted imaging).

MRI showed hyperintense signals in the bilateral hippocampus (**Figure 1**) and leptomeningeal enhancement in the left cerebral hemisphere (**Figure 2**). During hospitalization, comprehensive blood investigations revealed no significant abnormalities in thyroid function, rheumatoid factor, antistreptolysin O, antineutrophil cytoplasmic antibodies, lupus anticoagulant, infectious disease serologies, liver and kidney function, glycolipid metabolism, folate and vitamin levels, or tumor markers. Because AE was suspected, computed tomography scans of the chest, abdomen, and pelvis were performed and showed no evidence of malignancy. Cerebrospinal fluid (CSF) analysis revealed clear and colorless fluid with an opening pressure of 190 mmH_2_O, a leukocyte count of 39 cells/µL (9% lymphocytes, 91% polymorphonuclear cells), and a mildly elevated protein level (0.64 g/L). CSF cryptococcal antigen testing and cultures for bacteria, fungi, and tuberculosis were all negative. In addition, next-generation sequencing (NGS) of the CSF was negative, with no viral pathogens detected.

**Figure 3:**
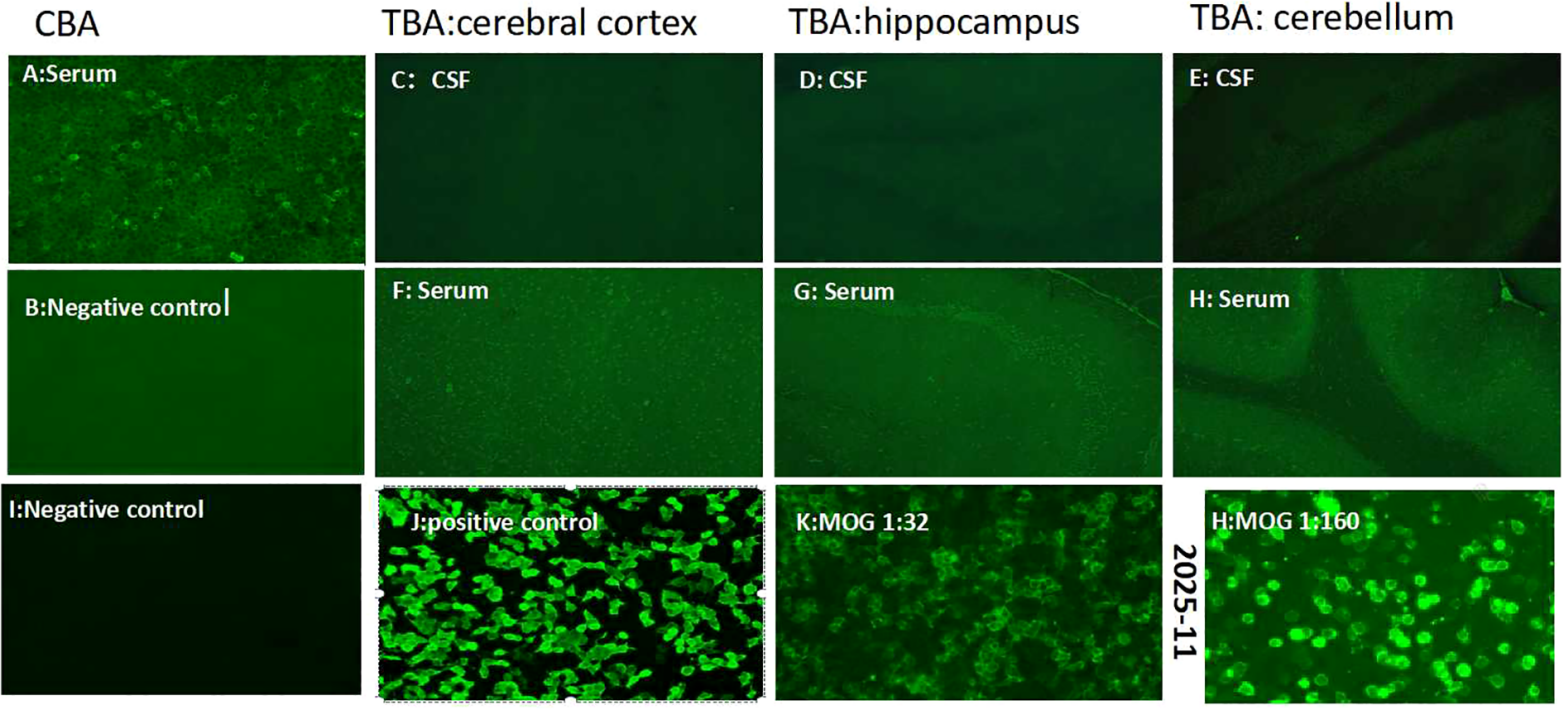
Detection of neurexin-3α antibody by cell-based assay in the patient’s serum (titer 1:10) **(A)**; tissue-based assay (TBA) demonstrates immunoreactivity of serum antibodies in the rat cerebral cortex, hippocampus, and cerebellum **(F–H)**. Myelin oligodendrocyte glycoprotein (MOG) antibodies (titer 1:32) were detected in the serum **(K)**. MOG antibodies with a titer of 1:160 were identified in the serum in November **(H)**.

Antibodies associated with AE, paraneoplastic syndromes, and demyelinating disorders, including NMDAR, AMPAR1, AMPAR2, LGI1, CASPR2, GABABR, DPPX, IgLON5, GlyRα1, GABAARα1, GABAARβ3, GABAARγ2, mGluR5, mGluR1, D2R, neurexin-3α, AK5, KLHL11, gAChR, CaVα2δ, Hu, Yo, Ri, CV2/CRMP5, amphiphysin, Ma2, Tr (DNER), Zic4, SOX1, GAD65, PKCγ, recoverin, titin, AQP4, GFAP, MBP, AQP1, and flotillin-1/2, were tested in serum and CSF using fixed cell-based assays (CBAs) with immunofluorescence double staining. MOG antibodies were assessed in serum and CSF using live CBAs. Initial screening was performed at dilutions of 1:10 for serum and 1:1 for CSF, and positive samples were further evaluated using serial 10-fold dilutions. Antibody confirmation in serum and CSF was carried out using tissue-based assays (TBAs) on rat brain sections at a dilution of 1:100. All analyses were conducted at Hangzhou Hongwang Clinical Laboratory in China. CBA revealed a 1:10 positive titer for neurexin-3α antibodies in serum, whereas TBA demonstrated positive neuronal membrane immunofluorescence in the hippocampus, cerebellum, and cerebral cortex. Neurexin-3α antibodies were not detected in the CSF. In addition, live CBA identified a positive titer of 1:32 for MOG antibodies in the serum, whereas CSF testing was negative (**Figure 3**).

Upon admission, the patient was treated with intravenous methylprednisolone at a dose of 500 mg daily for 5 days, followed by 250 mg daily for the next 5 days. Intravenous immunoglobulin was initiated on hospital day 4 at a dose of 400 mg/kg/day for 5 days. For seizure management, the patient received midazolam, sodium valproate, and levetiracetam, which progressively controlled epileptic activity. Olanzapine was administered to address persecutory delusions and agitation.

Despite adequate seizure control, the patient continued to exhibit recent memory impairment and altered mental status. Neurological evaluation at admission indicated cognitive dysfunction, with a Mini-Mental State Examination (MMSE) score of 16 and a Montreal Cognitive Assessment (MoCA) score of 13. After discharge, the patient was maintained on oral sodium valproate 500 mg twice daily, levetiracetam 750 mg twice daily, clonazepam 1 mg twice daily, and olanzapine 2.5 mg once daily at bedtime. At the 1-month follow-up, there was improvement in cognitive performance, with MMSE and MoCA scores increasing to 20 and 15, respectively.

In November, the patient experienced occasional epileptic seizures triggered by strong auditory stimuli. The treatment regimen was adjusted at a local hospital to include oral sodium valproate 500 mg twice daily, levetiracetam 750 mg twice daily, lacosamide 150 mg twice daily, perampanel 2 mg, and clonazepam 1 mg once daily at bedtime. Live CBA showed a positive serum titer of 1:160 for MOG antibodies. Rituximab-based immunosuppressive therapy was initiated, consisting of intravenous infusions of 100 mg and 500 mg, with subsequent doses planned at 24 weeks and a final dose at 1 year. At the 3- and 5-month follow-up, the patient remained neurologically stable, with no evidence of aphasia or psychosis. Rituximab was well tolerated, and no adverse events were reported during follow-up. Following treatment, the patient exhibited marked improvements in memory function and successfully returned to work.

## Discussion

Neurexin-3α and MOG antibodies were detected in the serum of the patient. In recent years, the coexistence of multiple neural autoantibodies in a single patient has been increasingly reported. Among these, MOG and N-methyl-D-aspartate receptor (NMDAR) antibodies are most frequently identified together in cases of demyelinating disorders and/or encephalitis over the past decade (4, 5). The concurrent occurrence of encephalitis and demyelinating disease suggests a possible shared pathophysiological mechanism between these conditions. One hypothesis is that the inflammatory milieu generated during demyelination may trigger the production of anti-NMDAR antibodies, potentially leading to encephalitis (6). Alternatively, the presence of anti-NMDAR antibodies might increase susceptibility to demyelinating lesions, possibly through complement activation or other immune-mediated mechanisms (7). In addition, shared genetic or environmental risk factors may play a role in the co-occurrence of these disorders (8).

The international MOGAD panel has established updated diagnostic criteria for MOG antibody–associated disease (MOGAD), highlighting MOG-IgG as a core diagnostic requirement for diagnosis. Patients who present with core clinical phenotypes—such as optic neuritis, myelitis, acute disseminated encephalomyelitis (ADEM), monofocal or polyfocal cerebral deficits, brainstem or cerebellar syndromes, or cerebral cortical encephalitis (often accompanied by seizures)—and demonstrating unequivocally positive MOG-IgG in serum via fixed or live CBAs can be diagnosed with MOGAD (9). Recent evidence indicates that MOGAD may also manifest as aseptic meningitis, characterized by leptomeningeal enhancement on neuroimaging (10–13). Although leptomeningeal enhancement occurs in 2–15% of MOGAD patients, it may serve as the initial presenting feature in some cases.

Cryptococcal antigen testing and NGS of the CSF revealed no evidence of infectious agents. In this case, the patient presented with prodromal symptoms of infection, including sore throat, rhinorrhea, cough, and fever. Since neurexin-3α antibody-mediated encephalitis is often preceded by infectious-like prodromal features, some scholars have proposed that it may represent a form of post-infectious encephalitis (14). The clinical syndrome, bilateral hippocampal MRI abnormalities, TBA confirmation, and favorable response to immunotherapy support a likely pathogenic role of neurexin-3α antibodies in this case, although the significance of low-titer serum-only positivity remains incompletely understood. Despite the presence of leptomeningeal enhancement in our patient, the current diagnostic criteria for MOGAD were not fulfilled. Consequently, we diagnosed the patient with concurrent anti-neurexin-3α encephalitis and potential anti-MOG-associated involvement.

A meta-analysis reported a recurrence rate of 63.4% in patients with MOG and NMDAR antibody overlapping syndrome (MNOS). The coexistence of both antibodies may contribute to this elevated recurrence risk (5). In the case, live CBA assay detected a positive titer of 1:160 for MOG antibodies in the serum during the second hospitalization. Given the potentially relapse rate in overlap syndromes compared to single-antibody disorders, rituximab was initiated in the case, resulting in a favorable clinical response.

We highlight the importance of comprehensive autoantibody screening—including testing for neurexin-3α antibodies—when AE is suspected, particularly in patients presenting with headache and gastrointestinal symptoms followed by acute-onset seizures and memory impairment. This case describes concurrent anti-neurexin-3α AE and,possible anti-MOG -associated involvement. thereby broadening the recognized spectrum of multi-antibody AE syndromes. In patients identified with anti-neurexin-3α encephalitis who develop optic neuritis, myelitis, ADEM, monofocal or polyfocal cerebral deficits, brainstem or cerebellar syndromes, or cerebral cortical encephalitis or leptomeningeal enhancement, evaluation for anti-MOG antibodies should be considered. Identification of dual antibody positivity may support more aggressive acute management and tailored long-term immunotherapy, which may contribute to improved clinical outcomes.

This study has several limitations. Polymerase chain reaction testing for herpes simplex virus, varicella-zoster virus, and enterovirus was not performed. The significant elevation of CSF leukocytes, with a predominance of polymorphonuclear cells (91%), was difficult to interpret. However, in the context of negative NGS results and the patient’s favorable response to high-dose corticosteroid therapy, a central nervous system infection was considered unlikely. Given the presence of fever and the established association between blood–brain barrier disruption and MOG-IgG–positive patients, we hypothesize that a preceding upper respiratory tract infection may have triggered an abnormal immune response, resulting in the generation of both neurexin-3α and MOG antibodies. Another limitation is the relatively short follow-up period of five months, which is insufficient to assess the long-term risk of disease relapse.

## Data Availability

The raw data supporting the conclusions of this article will be made available by the authors, without undue reservation.
